# Reported reasons for non-use of insecticide-treated nets in large national household surveys, 2009–2021

**DOI:** 10.1186/s12936-023-04490-w

**Published:** 2023-02-21

**Authors:** Hannah Koenker, E. Kuor Kumoji, Marcy Erskine, Robert Opoku, Eleanore Sternberg, Cameron Taylor

**Affiliations:** 1 Tropical Health LLP, London, UK; 2 Johns Hopkins Center for Communication Programs, Baltimore, USA; 3 International Federation of Red Cross and Red Crescent Societies, Geneva, Switzerland; 4 The DHS Program, ICF, Rockville, USA; 5 University of Antwerp, Medicine and Health Sciences, Antwerp, Belgium

## Abstract

**Background:**

Insecticide-treated nets (ITN) are the cornerstone of modern malaria vector control, with nearly 3 billion ITNs delivered to households in endemic areas since 2000. ITN access, i.e. availability within the household, based on the number of ITNs and number of household members, is a pre-requisite for ITN use. Factors determining ITN use are frequently examined in published literature, but to date, large household survey data on reasons given for non-use of nets have not been explored.

**Methods:**

A total of 156 DHS, MIS, and MICS surveys conducted between 2003 and 2021 were reviewed for questions on reasons why nets were not used the previous night, identifying twenty-seven surveys. The percent of nets that were reported used the previous night was calculated for the 156 surveys, and frequencies and proportions of reasons for non-use were calculated within the twenty-seven surveys. Results were stratified by household supply of ITNs in three categories (not enough”, “enough”, and “more than enough”) and by residence (urban/rural).

**Results:**

The proportion of nets used the previous night averaged over 70% between 2003 and 2021, with no discernible change over this period. Reported reasons for why a net goes unused fell largely into three categories—nets that are extra/being saved for future use; the perception that there is little risk of malaria (particularly in dry season); and “other” responses. Net attributes such as colour, size, shape, and texture, and concerns related to chemicals were the least frequent reasons given. Reasons for non-use of nets varied by household net supply, and in some surveys by residence. In Senegal’s continuous DHS, the proportion of nets used peaked during high transmission season, and the proportion of nets that went unused due to “no/few mosquitoes” peaked during the dry season.

**Conclusions:**

Unused nets were primarily those being saved for later use, or were not used due to perceived low risk of malaria. Classifying reasons for non-use into broader categories facilitates the design of appropriate social and behaviour change interventions to address the major underlying reasons for non-use, where this is feasible.

**Supplementary Information:**

The online version contains supplementary material available at 10.1186/s12936-023-04490-w.

## Background

Insecticide-treated nets (ITN) are the cornerstone of modern malaria vector control, with nearly 3 billion ITNs delivered to households in endemic areas since 2000 [1]. Consistent use of ITNs provides the most protection from malaria vectors, but households may only have enough nets for all household members in the several months immediately following mass ITN distributions after which the nets begin to wear out [2–5]. ITN access, i.e. availability within the household, determined by the number of ITNs and number of household members, is a prequisite for ITN use. ITN access is defined as the proportion of the population that could sleep under an ITN if each ITN in the household were used by up to two people. Once ITNs are in a given household, individuals may choose to use or not use them on a given night, with structural, cultural, opportunistic, ideational, and social barriers impeding optimal use [6–8].

Many papers evaluate determinants of ITN use, although not all account for ITN access. A primary factor influencing use of available nets is the perceived risk of malaria, due to seasonality of transmission; ITN use among those with access is typically lower during long, hot, dry seasons, when malaria vectors and other nuisance biting insects are less abundant [9]. Perceptions of heat and feeling closed in are frequently cited alongside each other [6, 10]. ITN use is also affected by who within a given household can share a single ITN based on age, cultural and social norms, as well as space available to hang ITNs [11–14]. Children under 5 years of age and women of reproductive age are consistently prioritized for ITN use, particularly when households do not have enough ITNs, with adolescents (especially boys) the least prioritized [15]. The condition of an ITN, related to its age and the development of holes and tears, is associated with early discarding of ITNs and, therefore, lack of use [16–19]; the decay rate of ITNs is a critical component of overall trends in ITN access, determining how quickly coverage declines following mass distribution campaigns and other large-scale distributions [2, 5].

Pulford et al. [6] reviewed 22 available studies in 2011 for reasons why nets went unused, finding that discomfort due to heat and perceived low risk of malaria due to low mosquito density were the primary reasons cited, but noted that findings were tentative given the dearth of published studies. Since this time, large national household surveys including Malaria Indicator Surveys (MIS) and Demographic and Health Surveys (DHS) have in several cases added questions about reasons for not using nets. This paper summarizes the available MIS and DHS data and explores trends in ITNs use. Finally, recommendations are given for further exploration of reasons for non-use of ITNs.

## Study objectives

The study objective is to use national population-based household survey data to characterize the reasons underlying non-use of ITNs. The goal of the analysis is to explore the reasons for not using an ITN during the previous night in relation to net supply at household level, and how these reasons vary by country and, where possible, by time. The research questions are:

1. What proportion of nets were used the night prior to the survey?
2. Of nets that went unused, what are the most frequently reported reasons for non-use, and how do reasons vary by country, household net supply, and residence?

## Methods

**Fig. 1:**
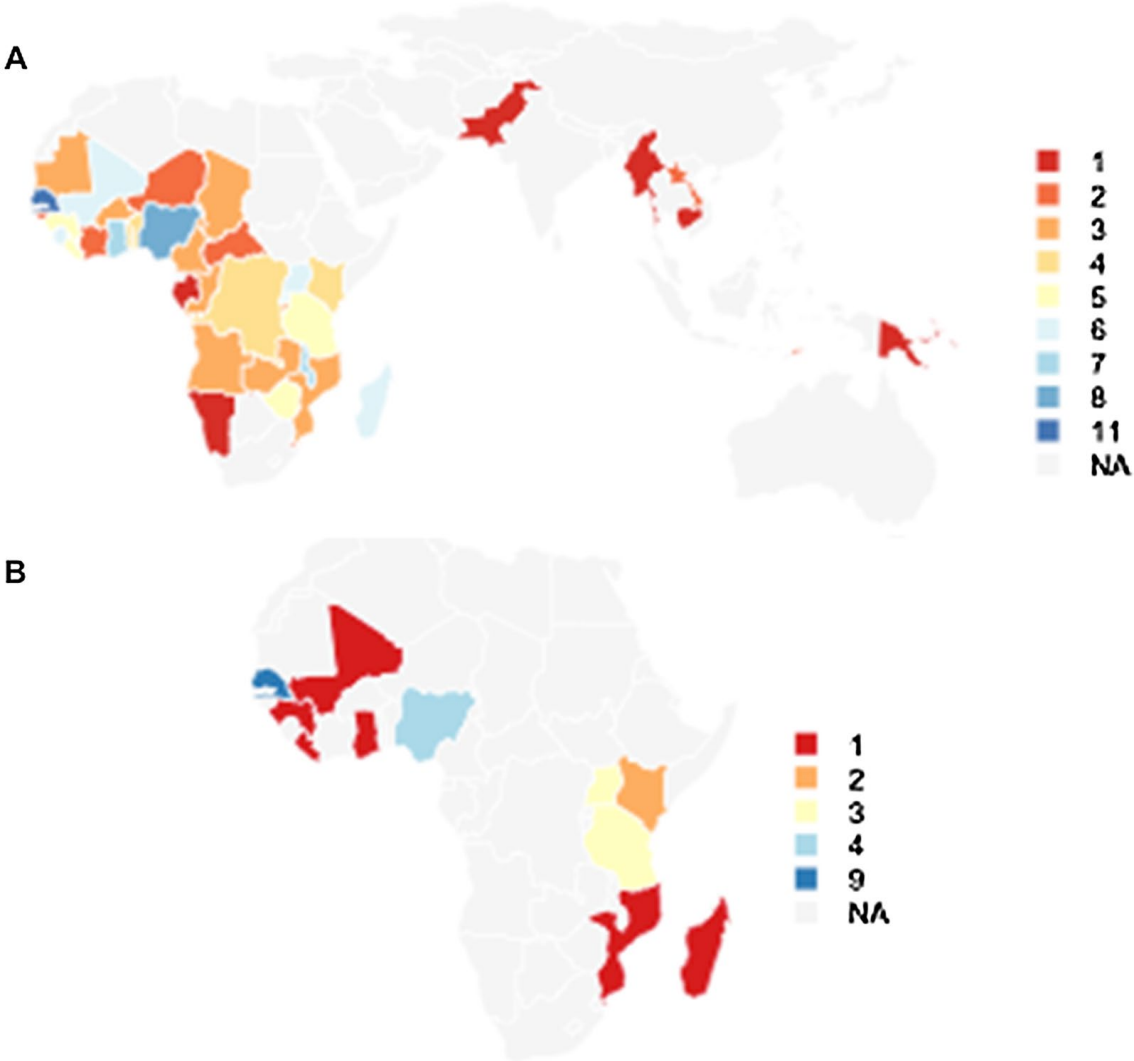
**A** Number of DHS, MICS, MIS surveys per country included in study objective 1; **B** Number of surveys per country containing questions about the reasons nets were not used the previous night

For the first study objective, 156 DHS, MIS, and Multiple Cluster Indicator Surveys (MICS) survey data collected since 2003 were downloaded with permission from dhsprogram.com and mics.unicef.org (Fig. 1A). Each dataset was reshaped to a long format to create a net file with details including its age, whether it was an ITN, the number of users, and whether it was reported to have been used the previous night. The Roll Back Malaria indicator for the percentage of nets used the previous night (out of all nets observed in the survey) was calculated for all surveys and linear regression was used to assess temporal changes for each type of survey. To evaluate net use in the context of household ITN supply, a variable was created according to ITN supply levels where “not enough” indicated less than 0.5 nets available per person (less than one ITN per two people), “enough” indicated 0.5 to 0.75 nets available per person (at least one ITN per two people), and “more than enough” nets indicated a supply of 0.75 or more nets available per person (i.e. at least two nets per three people). Households consisting of one person with one net were categorized as “enough”, rather than “more than enough”. For this variable, both untreated nets and ITNs were included.

For the second study objective, all DHS and MIS surveys were reviewed and twenty-seven published surveys from eleven countries (Fig. 1B) from 2009 or later were identified as having included a follow-up question for unused nets, asking for the reason or reasons that the net was not used (in eight surveys, multiple responses were possible, while in nineteen, only a single response could be selected). Sixteen were Malaria Indicator Surveys conducted during peak malaria transmission season (approximately three months of fieldwork), and eleven were DHS surveys including the Madagascar 2021 DHS, Nigeria 2018 DHS, the Tanzania 2015–16 DHS/MIS, and eight continuous DHS surveys from Senegal (2011–2019). The DHS surveys also aligned with peak malaria transmission season but were conducted over a longer time period (up to 10 months in Senegal). Senegal surveys between 2008 and 2019 also included specific questions about households’ use of nets all year round and reasons why nets were not used all year round. The “svy” family of commands in Stata 17 was used to appropriately weight results within each country. Plots were produced with R.

## Results

**Fig. 2:**
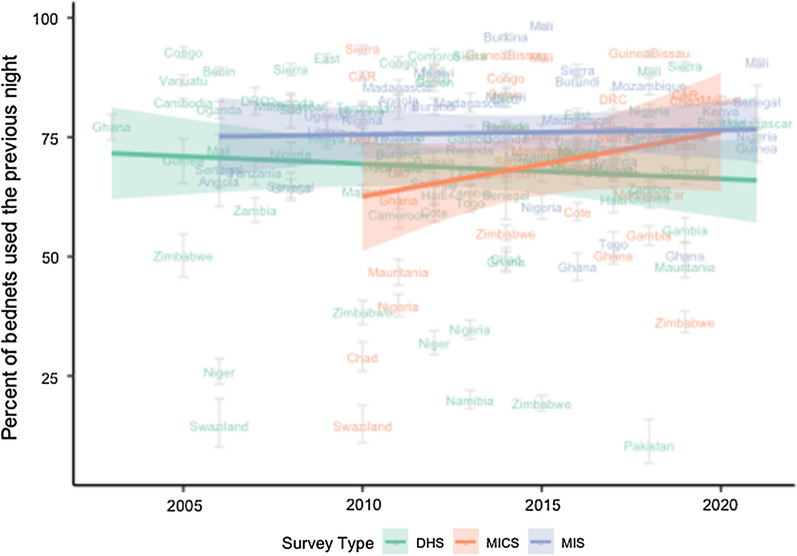
Percentage of nets used the night before the survey with linear trend: DHS, MICS, MIS surveys 2003–2020

The percent of nets used the previous night averaged 70.6% across all available (n = 156) DHS, MIS, and MICS surveys since 2003 (Fig. 2). Linear regression stratified by survey type indicated that there was no significant change over time in the percentage of nets used the previous night for MIS (p = 0.822), DHS (p = 0.499) or MICS (p = 0.181). MIS surveys, conducted during high transmission season, were associated with a 7.3-point increase in rates of net use when compared to DHS surveys (p = 0.027), which are generally conducted during dry season when malaria transmission is lower. Net use rates in MICS surveys did not differ significantly from DHS surveys (p = 0.931).

**Fig. 3:**
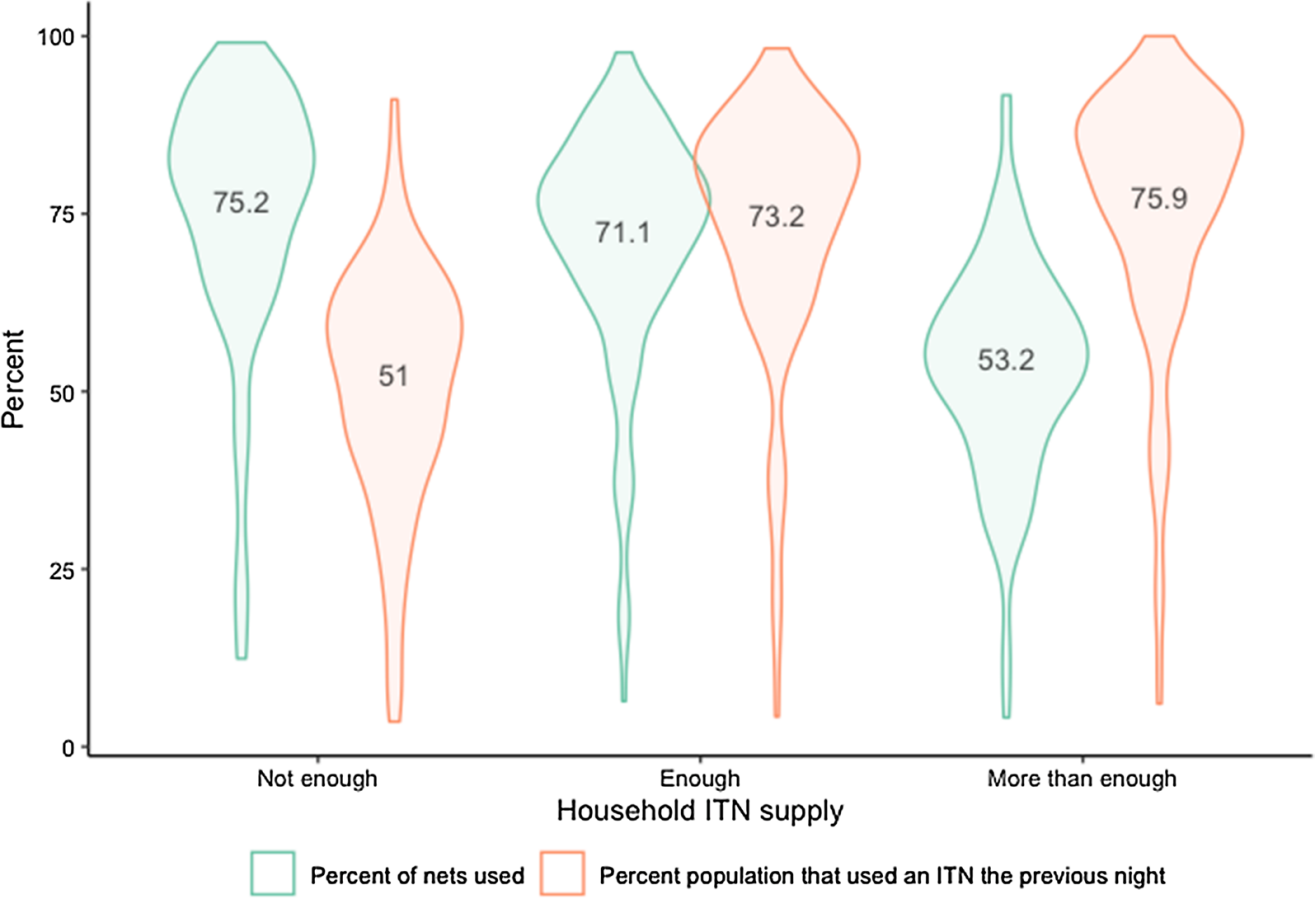
Violin plots with means for ITNs used the previous night and population use of ITNs, by household net supply level

The percent of nets used the previous night was 75.2% for households with not enough nets (at least one, but less than one net for two people) and 71.1% for households with at least one net for two people (“enough nets”) but less than two nets for three people (net:person ratio between 0.5 and 0.75), shown in Fig. 3. In contrast, in households with at least two nets for three people (“more than enough”) 53.2% of nets were used, potentially reflecting excess nets within the household or different net use behaviours by households with excess nets. Nonetheless, in these same households with “more than enough” nets, the percent of individuals using an ITN the previous night was 75.9%, on par with those living in households with sufficient ITNs (73.2%). For people living within households owning at least one but not enough ITNs, population ITN use was 51%.

## Reported reasons for not using nets

While the question “Why was this net not used the previous night” was consistent across all surveys, response options were inconsistent between countries and sometimes changed over time within a given country. Table 1 summarizes the response options and categorizes them into seven broad categories. The ‘other’ category captured ‘other’ responses (no survey had an “other, specify” option), as well as ‘not hung’ and ‘net not needed last night’, as the latter two responses fail to provide useful information about the respondent’s reasoning and thus cannot be grouped into other categories.

**Table 1:** Categorization of reasons why nets were not used

Category	Answer options from MIS
Extra	Extra; saving for later; stored away
Fears	Chemicals not safe; net is bad for health; superstition/witchcraft
Net attributes	Too rough/hard; too small; don’t like colour/shape/size; prefer other method
Objective	Too old/torn/dirty; no place to hang; usual user didn’t sleep here; net being washed; too weak/difficult to hang
Risk perception	No mosquitoes; no malaria; saving for rainy season
Subjective	Too hot; don’t like smell; feel closed in/afraid; no longer kills/repels mosquitoes; child doesn’t like; net never used; causes itching/coughing; brought bedbugs; slept outdoors
Other	Not needed last night; not hung; other; don’t know

**Fig. 4:**
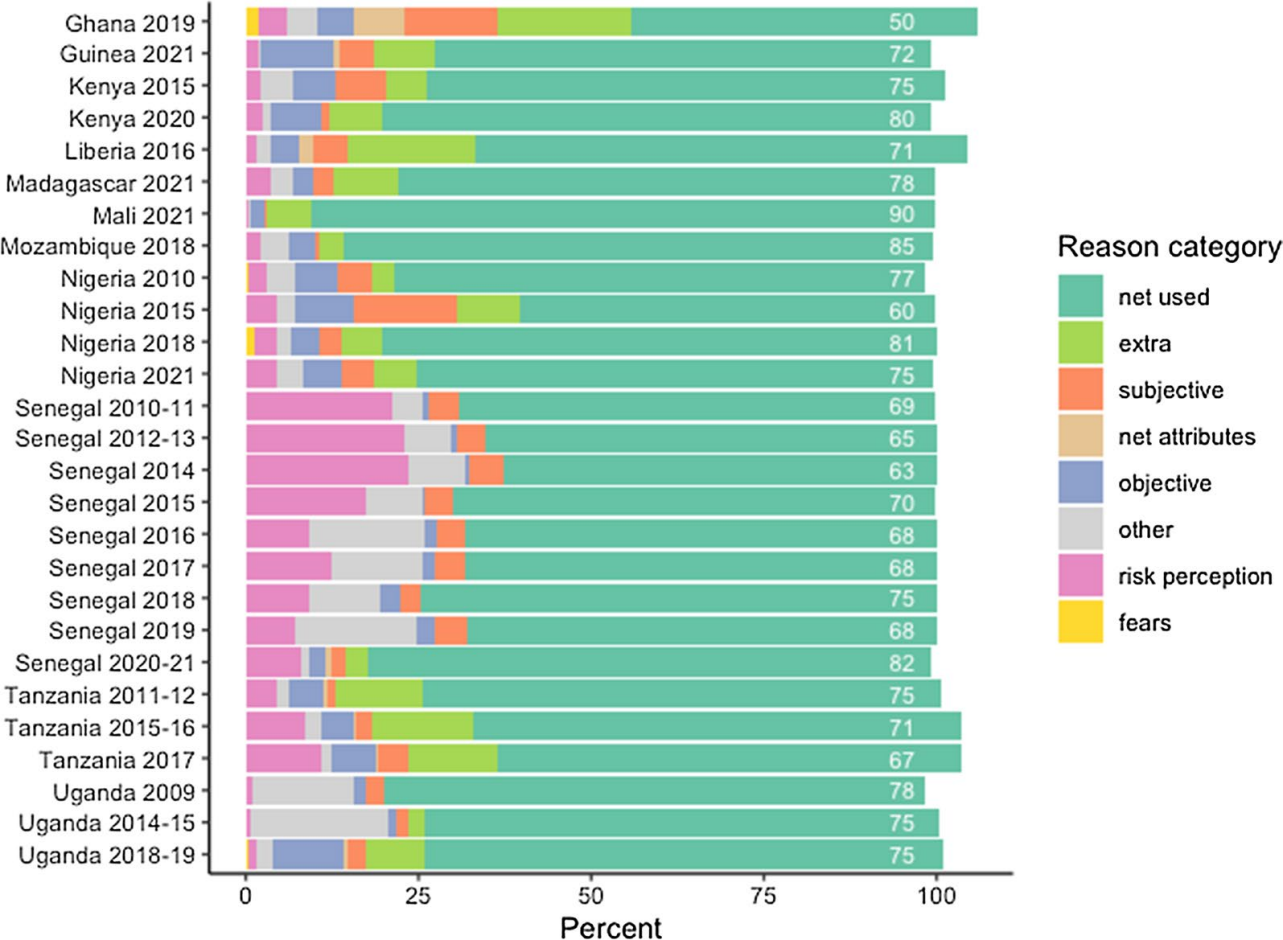
Distribution of reasons nets were not used, across surveys. Surveys allowing multiple responses total more than 100% of all nets in the survey

Figure 4 shows the percentage of nets used the previous night across twenty-seven surveys in eleven countries, and the reasons why certain nets were not used. The percentage of nets reported used ranged from 50% in Ghana 2019 to 85% in Mozambique 2018. Senegal’s continuous DHS surveys had the highest percentages of reasons relating to risk perception, with up to 25% of nets going unused due to “no mosquitoes” or “no malaria”. This category was relatively infrequent in other surveys, except for Tanzania where up to 11% of nets were unused due to risk perception, especially in lower-transmission areas (Additional file 1:, Fig. S1). “Extra/saving for later” nets were reported most frequently in Ghana 2019 (19%), Liberia 2016 (19%), and in Tanzania (12–14% of nets across the three available surveys). Senegal and Uganda had the highest rates of ‘other’ responses. In 2018, Uganda updated response options for this question to be more detailed, resulting in the ‘extra’ and ‘objective’ categories becoming more prominent; ‘extra’ category was comprised largely of ‘saving to replace other net’, while ‘objective’ was a combination of ‘usual user not here’ and ‘too old/torn’ (Additional file 1: Fig. S29). Subsequent surveys in Ghana, Guinea, Mozambique, Madagascar, Mali, and Nigeria adopted similar answer options.

**Fig. 5:**
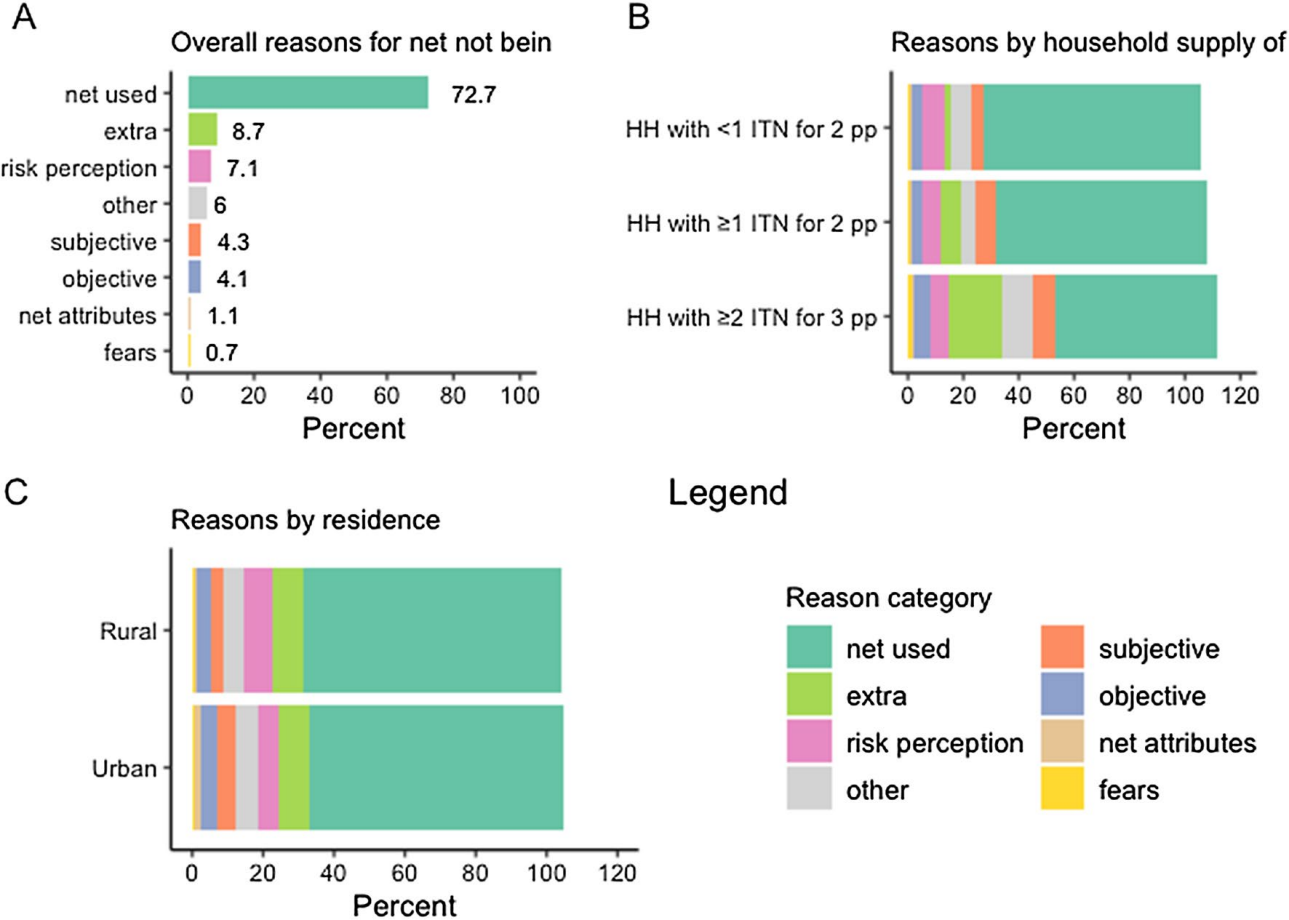
Summary of reasons nets were not used the previous night across surveys, by household supply of ITNs

Figure 5A summarizes the categories of reasons for non-use across all twenty-seven surveys, demonstrating that an average of 72.7% of nets were used the previous night, and that the leading category for non-use was “extra”, followed by “risk perception”. The least frequent categories cited as reasons for nets not being used were “net attributes” and “fears”. Figure 5B presents reasons for nets not being used in the context of household net supply.

Not surprisingly, “extra” nets comprised a higher proportion of reasons for non-use for nets in households with at least two ITN for three people (19.5%) compared to nets in households with fewer than one ITN per two people (2.8%) or nets in households with at least one ITN for two people but fewer than two nets for three people (7%). “Other” responses were more frequent for nets in households with at least two ITNs for three people (10.6% vs 5.2% and 7.1%), indicating that ‘other’ reasons are likely related to having extra nets, particularly in surveys prior to 2018 when response options did not capture extra nets well. The “risk perception” category was stable across ITN supply categories, ranging between 6.8% and 7.9%, as were “subjective” reasons, ranging from 7.7% to 8.4%. Reasons for non-use related to net attributes or fears comprised less than 2% of nets across all ITN supply categories.

When stratifying net use by residence (Fig. 5C), there was little difference overall between urban and rural settings, at 71.8–73.1% of nets used the previous night, respectively. Among the 156 available surveys, 33% showed no difference in net use by residence, while 27% had higher rates of net use in rural areas, and 40% had higher rates in urban areas. Further details on the magnitude of these differences across countries and surveys is provided in Additional file 1: Fig. S2.

Rural areas more frequently cited risk perception reasons for non-use, for 7.8% of nets, compared to urban settings (5.8% of nets), but cited subjective reasons less frequently (3.6% of nets in rural and 5.4% of nets in urban areas). More variation was observed in specific countries, however (Additional file 1: Figs. S3–S29). The urban/rural discrepancy in percentage of nets used was greatest in Ghana (37.4% in urban vs 58.8 in rural areas). Nets in urban areas of Nigeria were also consistently used less than those in rural areas in the four available surveys, declining from a 15.3 percentage-point difference in 2010 to a 6.1 percentage-point difference in 2021. In Kenya, Liberia, Madagascar, Mali, Mozambique, and Uganda, there was virtually no difference, however, and in Tanzania more urban nets were used than rural ones. Results were variable over the nine Senegal surveys. Urban residents in Ghana much more frequently reported “too hot” compared to their rural counterparts (20.7 vs 6.7 in 2019), as did respondents in Nigeria (2015, 2018, and 2021) and Liberia (2016), while the reverse was true in the three Tanzania surveys. Rural residents in Tanzania more frequently reported “no mosquitoes” than those in urban areas in all three surveys; the same was true in Senegal for surveys conducted from 2011 to 2016, but urban residents cited it more frequently in 2018 and 2021. Urban residents in Ghana (2019) also more frequently reported preferring other methods (for 9.9% of all nets observed), compared to rural areas (4.3% of all nets).

**Fig. 6:**
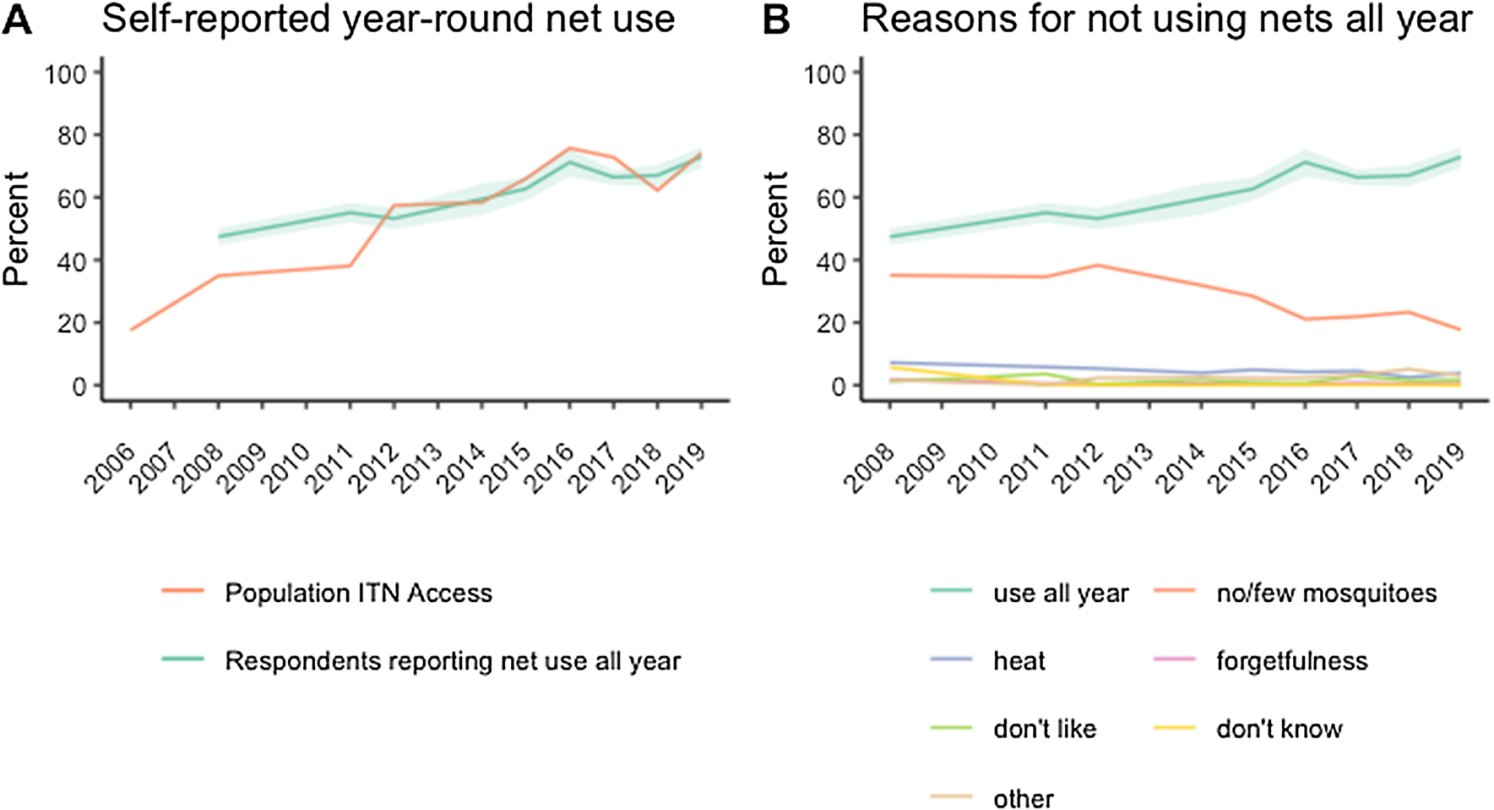
Proportion of households reporting year-round net use and households’ reported reasons for not using nets, Senegal 2008–2019

In eight surveys from Senegal two additional questions were asked. In households that owned at least one net, respondents were asked “do members of this household use nets all year round?” (Fig. 6A). The percentage of respondents reporting that people in their household did use nets year-round increased from 47.4% to 72.9% over the 2008–2019 period (p < 0.001 for trend) and generally tracked with increasing levels of population access to ITNs. In households responding “no”, a follow-up question was asked: “what are the reasons household members do not use nets year round?”. The most frequent answer was “no/few mosquitoes” (Fig. 6B), which fell from a high of 38.3% in 2012 to 17.7% in 2019, as a proportion of all households in the survey. “Heat” was the next most common response, ranging between 2.5 and 7.2%. Not liking the net and forgetfulness were relatively uncommon (less than 3.6% and 1.8% of all households in any survey, respectively).

**Fig. 7:**
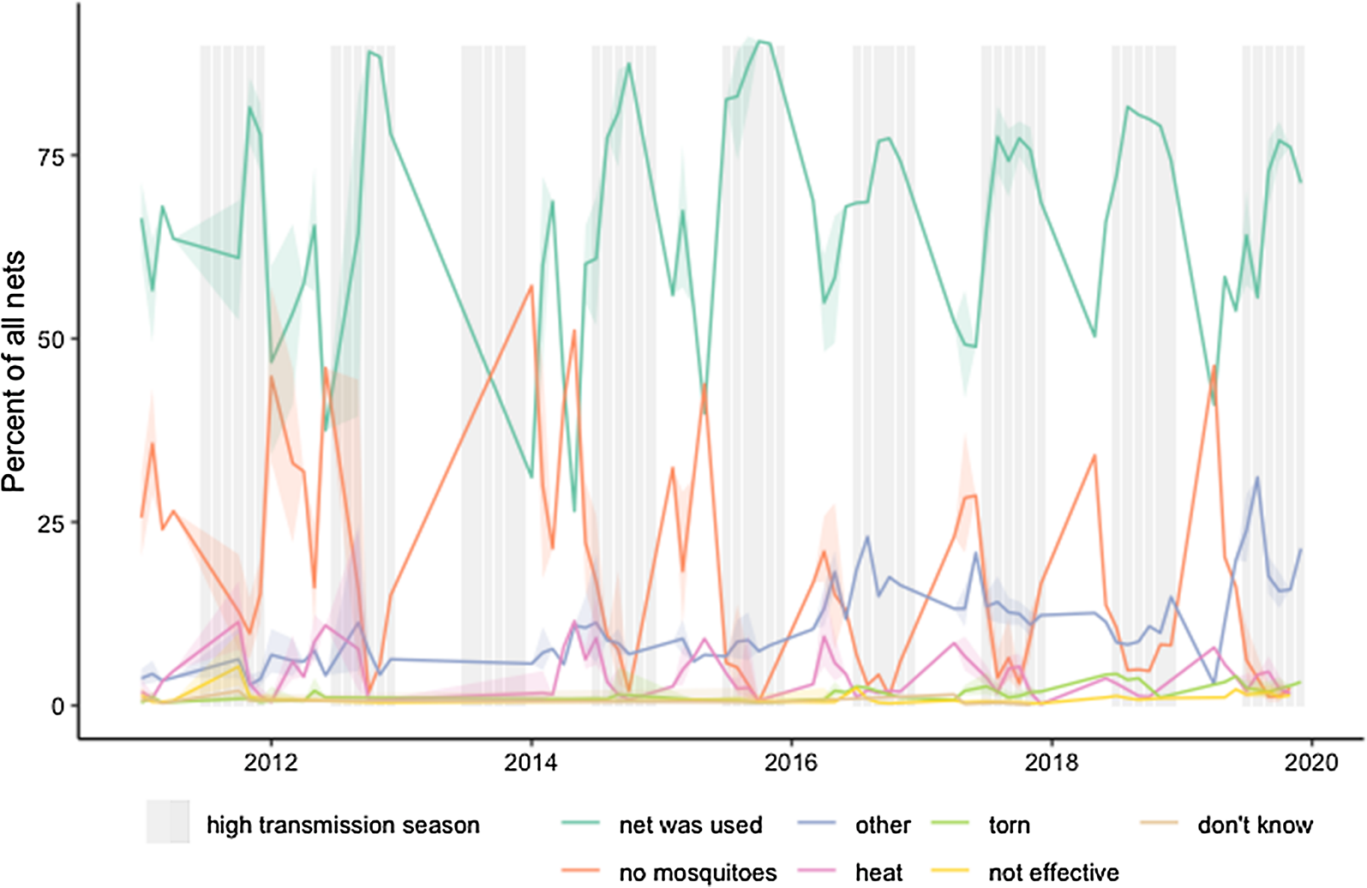
Percent of nets used the previous night (green) and reasons for non-use, Senegal 2011–2019

Seasonal trends in reasons for not using nets in Senegal were apparent (Fig. 7) with the percentage of nets used the previous night peaking during the high transmission season (shown approximatively above as July–December) and falling during the drier months of February-May. Correspondingly, the proportion of nets not used due to “no mosquitoes” peaked during the drier months.

## Discussion

Over the past nearly 20 years, an average of over 70% of ITNs were reported as being used the previous night in 156 large household surveys from forty-four countries. Questions about why nets go unused have only been included in twenty-seven surveys from eleven countries, but among these, the primary reasons given were that unused nets are surplus to immediate requirements or are not needed due to perceived low risk of malaria and/or mosquito bites. Responses related to extra nets were more frequent among households owning more ITNs than deemed strictly necessary by the World Health Organization (WHO) (one ITN per two people) [20]. Unsurprisingly, the proportion of nets used the previous night was lower in households with “more than enough” nets than in households with “not enough” or “enough” nets, while at the same time, the proportion of people that used an ITN the previous night was highest in households with “enough” or “more than enough” ITNs. Households with more than one ITN per two people may have acquired additional nets to cover individual sleeping spaces or to accommodate sleepers who cannot share a sleeping space and thus are able to have most household members sleep under a net; other households may have extra nets being saved for later use, when current nets wear out. Households with “not enough” ITNs had lower rates of population use, but high rates of nets being used—indicating that these households are using the nets that they have, and are challenged primarily by not having enough for other members of the family. It should be noted that having ‘extra’ nets is reflective of the inherent inefficiencies of ITN distribution systems, wherein some households will have too few while others may receive additional nets slightly earlier than required [2]. The authors view having extra nets on hand within households as a positive, given the unpredictability of net replacement timing.

Reasons related to net attributes, including size, shape, colour, texture, and mosquito-killing ability, were inconsistently included in survey questionnaires, but represented a negligible fraction of reasons for not using nets. While this does not preclude these issues from contributing to net non-use, it provides some evidence that these net attributes are not a key priority when families are making net use decisions. The 2011 Pulford review findings [6] that discomfort due to heat and perceived low mosquito density were the most widely identified reasons for non-use are partially confirmed here; heat per se was not widely reported in more recent surveys, but risk perception as a category, particularly for Senegal, was a key driver. Pulford et al. also use categories such as “social factors” (sleeping elsewhere), “technical factors” (not being able to hang a net), which are considered “objective” reasons for non-use in our study. Pulford’s review, conducted just as universal coverage campaigns were scaling up, was limited to 22 studies between 1990 and 2010. Since this time, a number of qualitative research studies have also been conducted, in which respondents cite being bothered by net attributes including smell, itching, shape, and size [13, 21–23]. However, these reasons were only rarely cited during quantitative surveys included in this study. Research from Senegal indicates that initial itchiness or smell of nets are transitory, noticeable when nets are first received, but subsiding over time, not impeding net use [22]. Other less preferable attributes of nets may similarly become less noticeable over time, and no longer constitute a key reason for non-use, particularly when, as in most countries distributing ITNs, there are seldom enough nets in good condition for everyone to use. Families thus face choices about using the imperfect ITNs they have, or risk contracting malaria.

Nearly 80 unique answer options were included across the surveys. The categorization of responses into “extra”, “risk perception”, “objective”, “subjective”, etc., is intended to facilitate interpretation and guide national malaria programmes and their partners in designing appropriate responses for improving net use. Where the majority of unused nets are not used due to subjective reasons, social behaviour change may be able to change attitudes and behaviours; however, where most nets are unused due to being too old or torn, programmes may need to focus on net maintenance behaviours and/or additional ITN distribution to improve ITN use rates.

As one example, Senegal has focused messaging over the last decade to address the perceived lower risk of malaria in the hot/dry season, in part because of findings in these surveys, through the “Trois Toutes” campaign (“Toute la famille, toutes les nuits, toute l’année” or “Every family member; every night; all year round”). Self-reported use of nets all year round has increased over time, although it remains unclear whether this is driven primarily by corresponding increases in overall access to ITNs with the household, or represent real changes in behaviour for more consistent ITN use. The continuous DHS in Senegal, conducted over multiple months annually for the last 8 years, present a unique opportunity for assessing trends over time in year-round use, as well as evaluating the associations between seasons and frequency of certain responses, notably “no/few mosquitoes”. Indeed, net use peaks during periods of high malaria transmission, while the proportion of nets not used due to “no mosquitoes” peaks during the hot dry season when mosquito densities are substantially lower.

Another example of refining the net non-use question to better inform programming is from Uganda. Following the 2009 MIS survey Uganda implemented “hang up campaigns” to ensure nets were hung and used, partially in response to low hanging rates observed in the 2009 and other surveys. Operational research showed that these hang up campaigns did not improve hanging or use rates, as net hanging increased at similar rates over time in control and intervention groups [24]. In its most recent surveys, Uganda teased apart the nebulous “not hung” answer option to better focus on specific barriers to net use, enabling the programme to understand what lies behind the non-use of nets. Key reasons for non-use in 2018 were “saving net for later”, “user not here”, and “too old/torn”, none of which are best addressed with social behaviour change (SBC) efforts to hang up nets. The specification of reasons for non-use enables programmes and their SBC partners to better design and target net use interventions. The absence of these types of questions even in many recent surveys has been a missed opportunity, particularly as ITNs remain the primary tool for malaria vector control across the globe. Happily, however, the question is now standard in MIS and DHS surveys conducted since 2019, with a set of recommended response options drawn in large part from earlier iterations of the present study.

Urban and rural differences in net use have been observed in many countries and settings, and typically reflect differences in perception of risk of malaria, driven by underlying malaria transmission rates (which can also vary considerably within urban areas [25]). In Ghana, Guinea, and Nigeria, where malaria risk is considerably lower in urban areas [26–30], nets are used at lower rates by urban residents, while in Tanzania, low-transmission zones tend to be more rural, contributing to lower net use rates in rural areas. In the context of lower malaria risk, people are more likely to cite subjective reasons for not using available nets, as the Ghana results demonstrate here (Additional file 1: Fig. S3).

These findings also highlight that there may be more limited “room for improvement” in ITN use than previously thought. Nets not used for ‘objective’ reasons and those that are ‘extra’ are relatively impervious to social behaviour change communication, and these categories explain non-use for, on average, 13% of all nets in the included surveys, with a range of 0.5% to 24.6% of all nets depending on the country and survey. Even with highly effective social behaviour change, not all nets can be reasonably expected to be used.

## Limitations

The study has several limitations. First, the question of reasons why nets were not used was included in only twenty-seven surveys in eleven countries, with nine of these from Senegal. While it is not possible to generalize reasons for non-use of nets to other countries, the present findings show that there are substantial similarities across countries in overall percentage of nets used and the relative importance of certain types of reasons. Second, response options and the number of reasons vary considerably by country, from seven in Senegal to seventeen in Liberia and Mozambique. Nonetheless, some of the differences in response options were minimal changes in wording, and major categories of reasons were generally included in each survey. Third, the categorization of the reasons for non-use into broader categories relies on assumptions about which barriers are similar, and opinions may differ depending on subject familiarity, lived experience, and other factors. Some reasons may also belong in multiple categories. Fourth, around half the surveys posed the question about reasons for why a net wasn’t used the previous night as a multiple choice question, while the other half restricted it to a single response. This may introduce some unequal weighting into the results, or put more emphasis on single-choice responses to the exclusion of other possible reasons for not using nets. Finally, there were a substantial number of responses recorded as “other” in many of the surveys; it cannot be determined what type of reason this may have been, although it seems likely that they are at least in part related to extra nets or saving nets for future use, given the increase in other responses among households with “more than enough” nets.

## Conclusion

The percentage of nets used the previous night averaged over 70% from 2003 to 2020, with no discernible change over this period. Reported reasons for why a net was unused fell largely into three categories—nets that were extra/being saved for future use; the perception that there was little risk of malaria (particularly in dry season); and “other” responses. Net attributes such as colour, size, shape, and texture, and fears related to chemicals were the least frequent reasons given. Classifying reasons for non-use into broader categories facilitates the design of appropriate SBC interventions to address the major underlying reasons for non-use, where this is feasible. Finally, national malaria programmes should take advantage of the inclusion of this question in future surveys to provide actionable data to inform SBC programming.

## Supplementary Information

**Additional file1: Fig. S1**: Reported net use and reasons for non-use by low and high transmission zones, Tanzania 2017-18 MIS. **Fig. S2**: Percentage-point difference in urban vs rural percent of nets used the previous night, over time. **Fig. S3**: Reasons nets were not used the previous night, Ghana 2019. **Fig. S4**: Reasons nets were not used the previous night, Guinea 2021. **Fig. S5**: Reasons nets were not used the previous night, Kenya 2015. **Fig. S6**: Reasons nets were not used the previous night, Kenya 2020. **Fig. S7**: Reasons nets were not used the previous night, Liberia 2016. **Fig. S8**: Reasons nets were not used the previous night, Madagascar 2021. **Fig. S9**: Reasons nets were not used the previous night, Mali 2021. **Fig. S10**: Reasons nets were not used the previous night, Mozambique 2018. **Fig. S11**: Reasons nets were not used the previous night, Nigeria 2010. **Fig. S12**: Reasons nets were not used the previous night, Nigeria 2015. **Fig. S13**: Reasons nets were not used the previous night, Nigeria 2018**. Fig. S14**: Reasons nets were not used the previous night, Nigeria 2021. **Fig. S15**: Reasons nets were not used the previous night, Senegal 2011. **Fig. S16**: Reasons nets were not used the previous night, Senegal 2012. **Fig. S17**: Reasons nets were not used the previous night, Senegal 2014. **Fig. S18**: Reasons nets were not used the previous night, Senegal 2015. **Fig. S19**: Reasons nets were not used the previous night, Senegal 2016. **Fig. S20**: Reasons nets were not used the previous night, Senegal 2017. **Fig. S21**: Reasons nets were not used the previous night, Senegal 2018. **Fig. S22**: Reasons nets were not used the previous night, Senegal 2019. **Fig. S23**: Reasons nets were not used the previous night, Senegal 2020-2021. **Fig. S24**: Reasons nets were not used the previous night, Tanzania 2011-12. **Fig. S25**: Reasons nets were not used the previous night, Tanzania 2015. **Fig. S26**: Reasons nets were not used the previous night, Tanzania 2017-2018. **Fig. S27**: Reasons nets were not used the previous night, Uganda 2009. **Fig. S28**: Reasons nets were not used the previous night, Uganda 2014. **Fig. S29**: Reasons nets were not used the previous night, Uganda 2019

## Data Availability

Code is available at https://github.com/hkoenker/Reasons. Data are publicly available from www.dhsprogram.com and from mics.unicef.org.

## Abbreviations

DHS: Demographic and Health Surveys
ITN: Insecticide-treated net
LLIN: Long lasting insecticidal net
MICS: Multiple indicator cluster Survey
MIS: Malaria Indicator Survey
SBC: Social behaviour change
WHO: World Health Organization
