# Supplementary material for: Reported reasons for non-use of insecticide-treated nets in large national household surveys, 2009–2021
Source: Malar J. 2023 Feb 21;22:61. doi: 10.1186/s12936-023-04490-w (PMC9942310; doi:10.1186/s12936-023-04490-w)
Supplement: Supplementary file 1 — Additional file1: Fig. S1: Reported net use and reasons for non-use by low and high transmission zones, Tanzania 2017-18 MIS. Fig. S2: Percentage-point difference in urban vs rural percent of nets used the previous night, over time. Fig. S3: Reasons nets were not used the previous night, Ghana 2019. Fig. S4: Reasons nets were not used the previous night, Guinea 2021. Fig. S5: Reasons nets were not used the previous night, Kenya 2015. Fig. S6: Reasons nets were not used the previous night, Kenya 2020. Fig. S7: Reasons nets were not used the previous night, Liberia 2016. Fig. S8: Reasons nets were not used the previous night, Madagascar 2021. Fig. S9: Reasons nets were not used the previous night, Mali 2021. Fig. S10: Reasons nets were not used the previous night, Mozambique 2018. Fig. S11: Reasons nets were not used the previous night, Nigeria 2010. Fig. S12: Reasons nets were not used the previous night, Nigeria 2015. Fig. S13: Reasons nets were not used the previous night, Nigeria 2018. Fig. S14: Reasons nets were not used the previous night, Nigeria 2021. Fig. S15: Reasons nets were not used the previous night, Senegal 2011. Fig. S16: Reasons nets were not used the previous night, Senegal 2012. Fig. S17: Reasons nets were not used the previous night, Senegal 2014. Fig. S18: Reasons nets were not used the previous night, Senegal 2015. Fig. S19: Reasons nets were not used the previous night, Senegal 2016. Fig. S20: Reasons nets were not used the previous night, Senegal 2017. Fig. S21: Reasons nets were not used the previous night, Senegal 2018. Fig. S22: Reasons nets were not used the previous night, Senegal 2019. Fig. S23: Reasons nets were not used the previous night, Senegal 2020-2021. Fig. S24: Reasons nets were not used the previous night, Tanzania 2011-12. Fig. S25: Reasons nets were not used the previous night, Tanzania 2015. Fig. S26: Reasons nets were not used the previous night, Tanzania 2017-2018. Fig. S27: Reasons nets were not used the previous night, U [file 12936_2023_4490_MOESM1_ESM.docx]

# Additional file


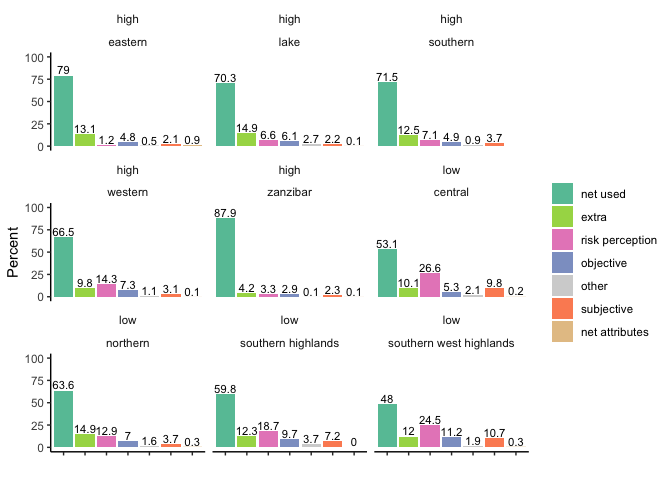


Fig. S1: Reported net use and reasons for non-use by low and high transmission zones, Tanzania 2017-18 MIS


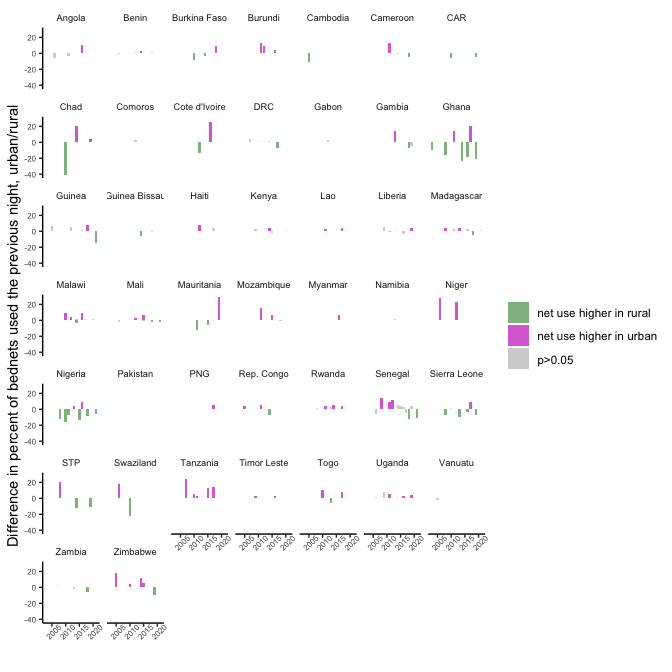


Fig. S2: Percentage-point difference in urban vs rural percent of nets used the previous night, over time

### Ghana


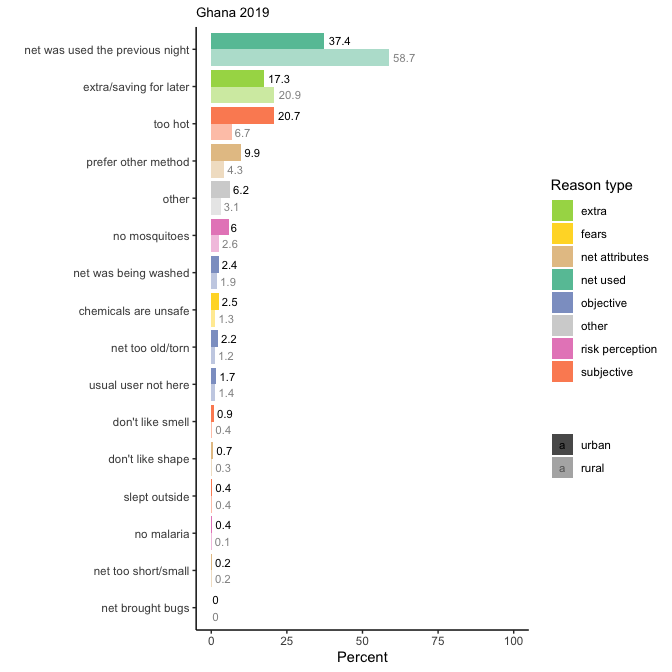


Fig. S3: Reasons nets were not used the previous night, Ghana 2019

### Guinea


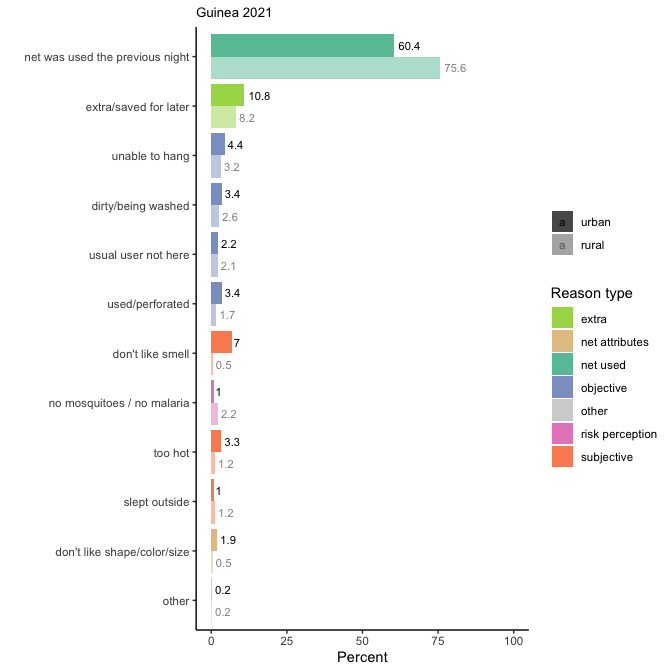


Fig. S4: Reasons nets were not used the previous night, Guinea 2021

### Kenya


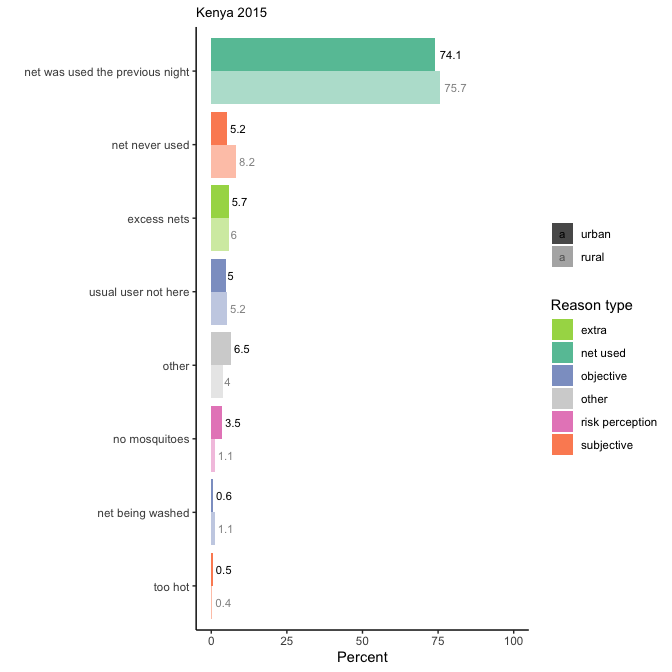


Fig. S5: Reasons nets were not used the previous night, Kenya 2015


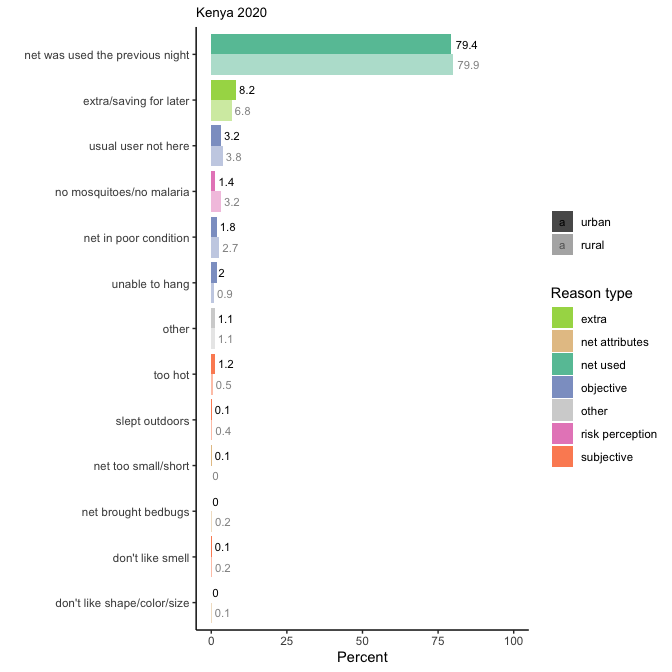


Fig. S6: Reasons nets were not used the previous night, Kenya 2020

### Liberia


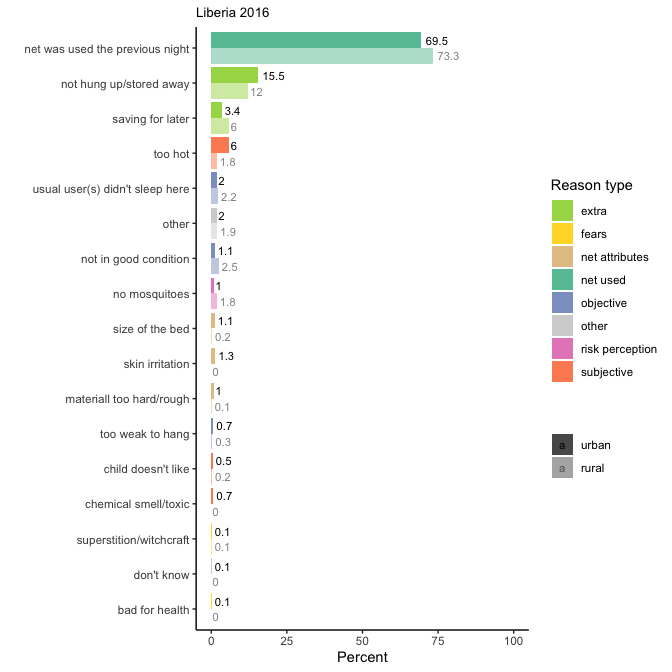


Fig. S7: Reasons nets were not used the previous night, Liberia 2016

### Madagascar


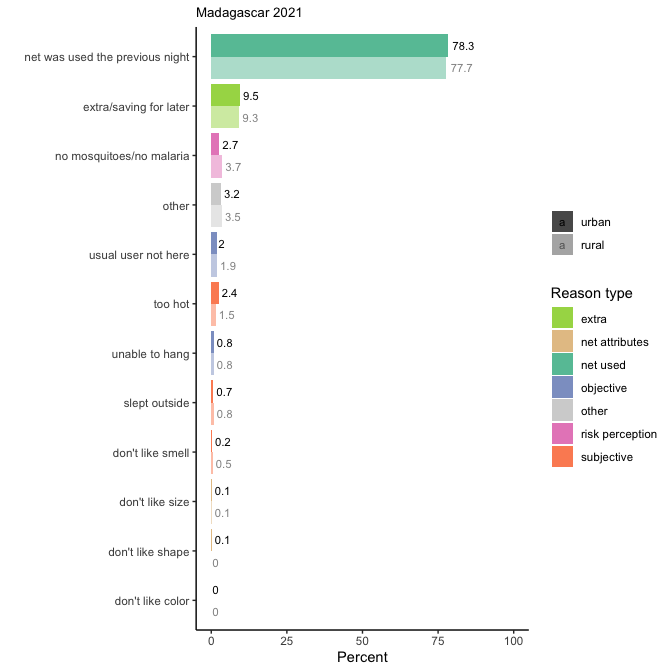


Fig. S8: Reasons nets were not used the previous night, Madagascar 2021

### Mali


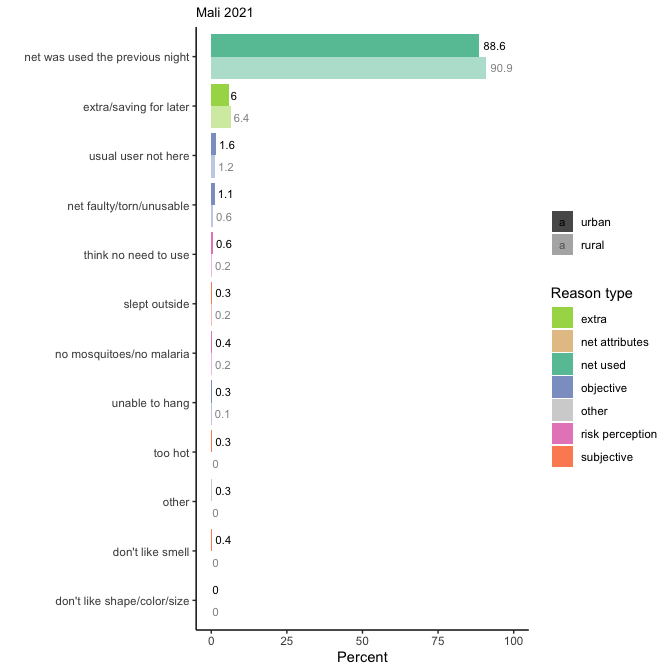


Fig. S9: Reasons nets were not used the previous night, Mali 2021

### Mozambique


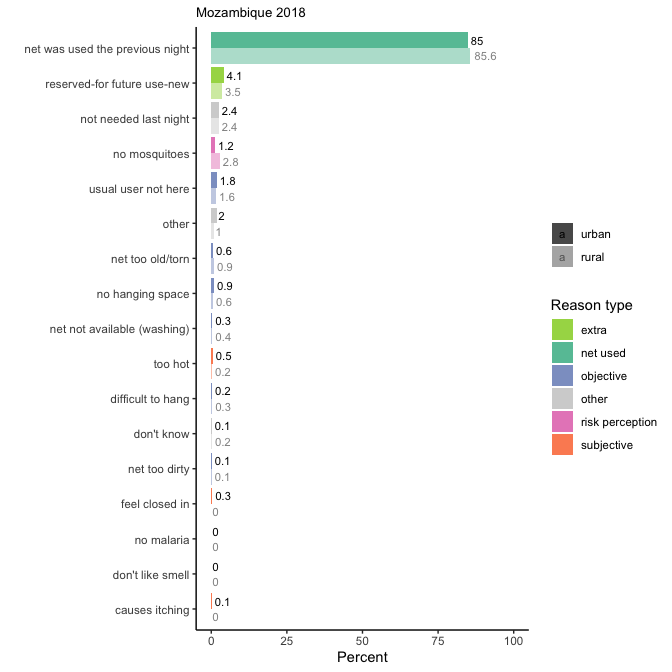


Fig. S10: Reasons nets were not used the previous night, Mozambique 2018

### Nigeria


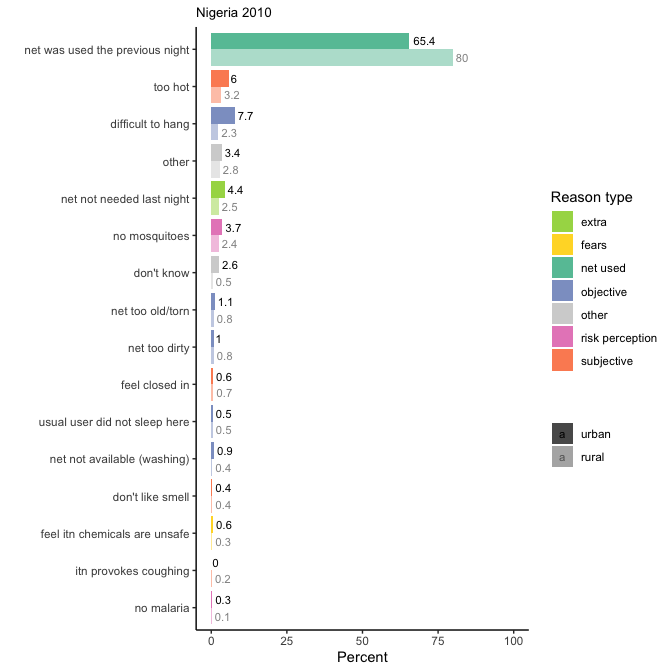


Fig. S11: Reasons nets were not used the previous night, Nigeria 2010


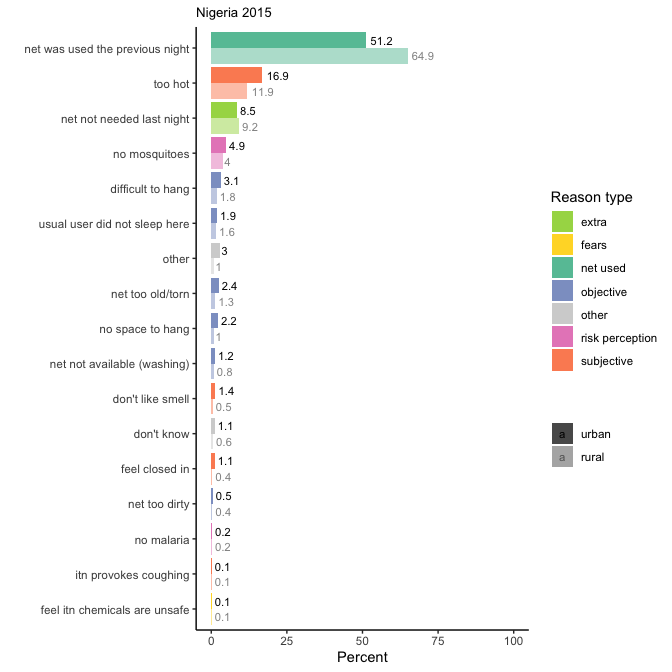


Fig. S12: Reasons nets were not used the previous night, Nigeria 2015


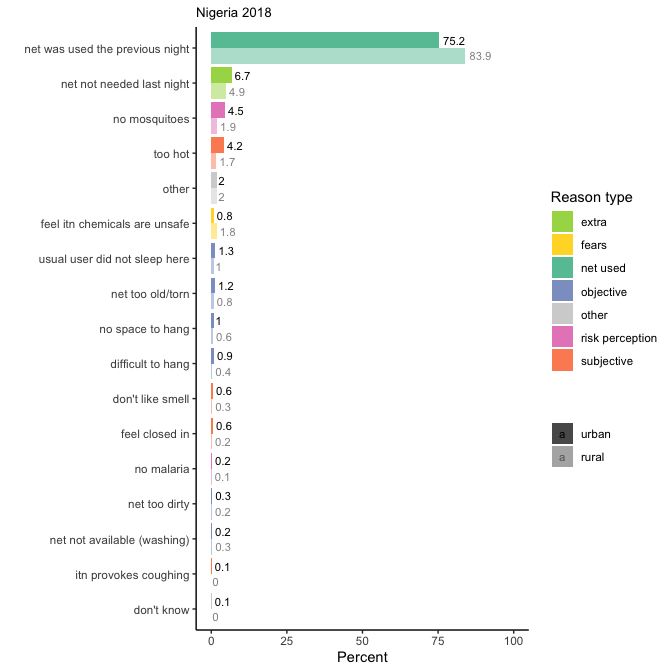


Fig. S13: Reasons nets were not used the previous night, Nigeria 2018


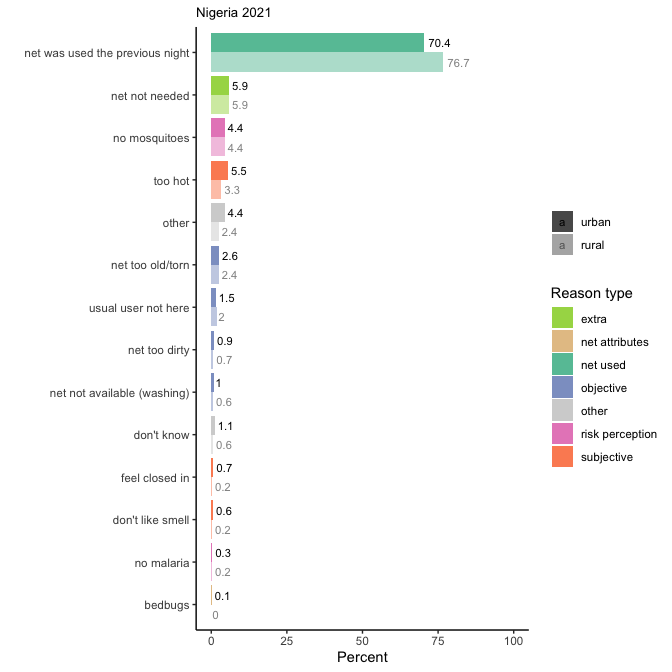


Fig. S14: Reasons nets were not used the previous night, Nigeria 2021

### Senegal


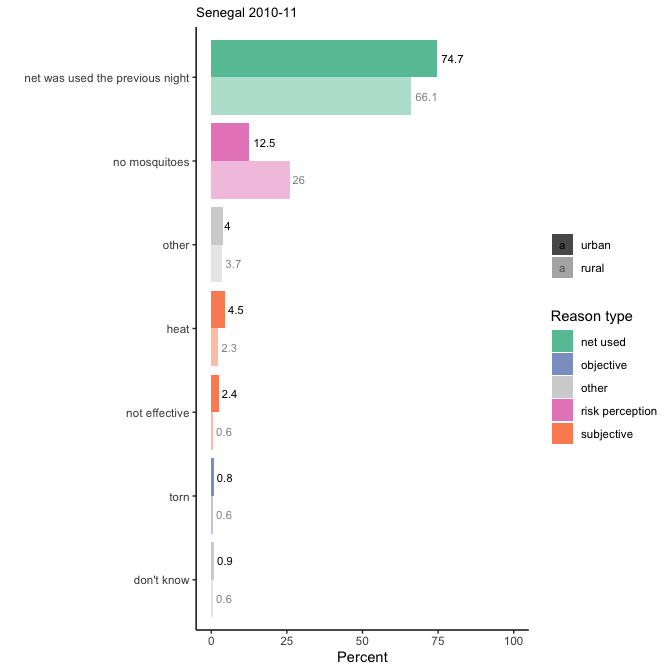


Fig. S15: Reasons nets were not used the previous night, Senegal 2011


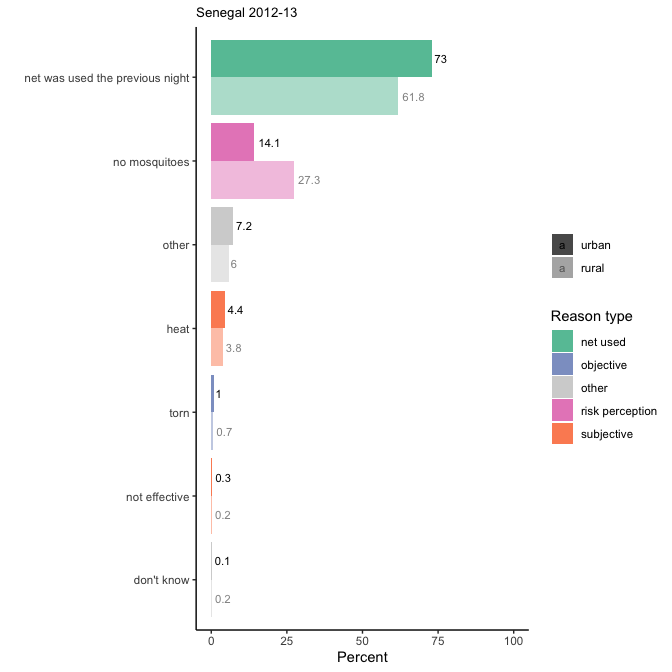


Fig. S16: Reasons nets were not used the previous night, Senegal 2012


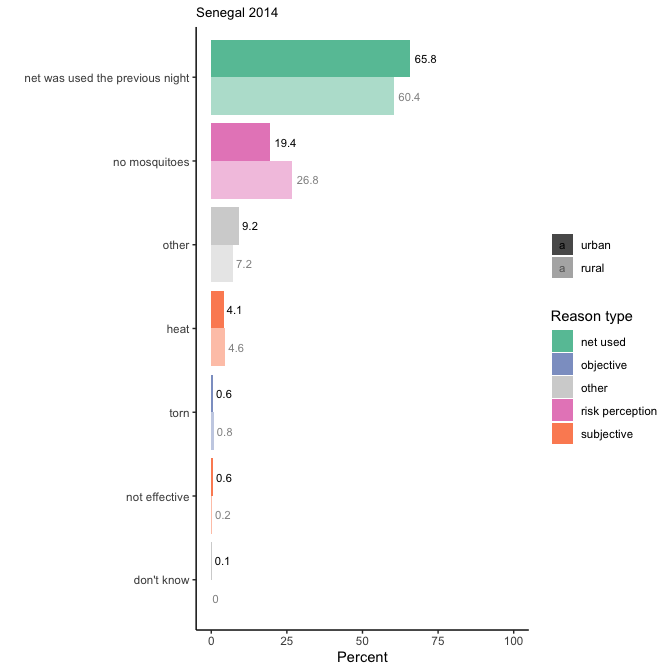


Fig. S17: Reasons nets were not used the previous night, Senegal 2014


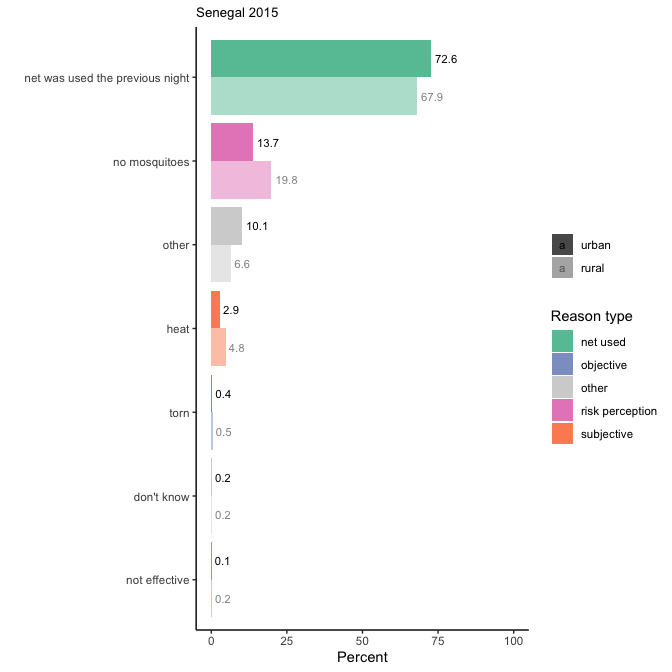


Fig. S18: Reasons nets were not used the previous night, Senegal 2015


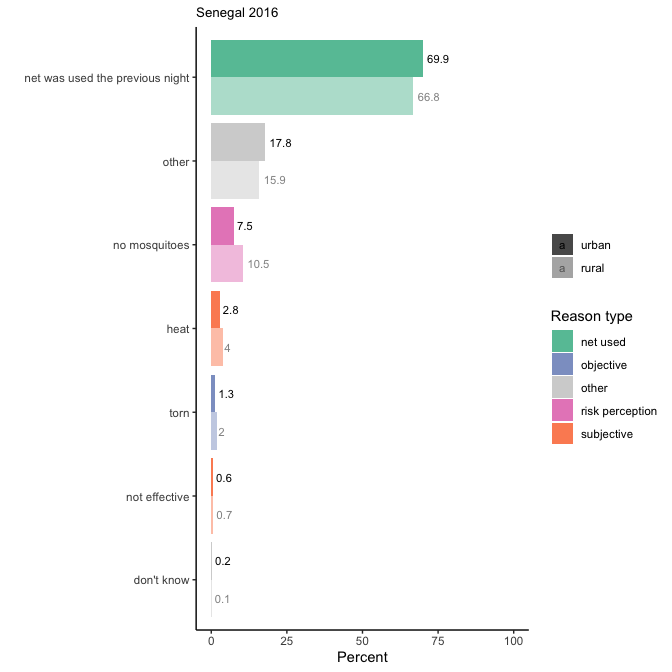


Fig. S19: Reasons nets were not used the previous night, Senegal 2016


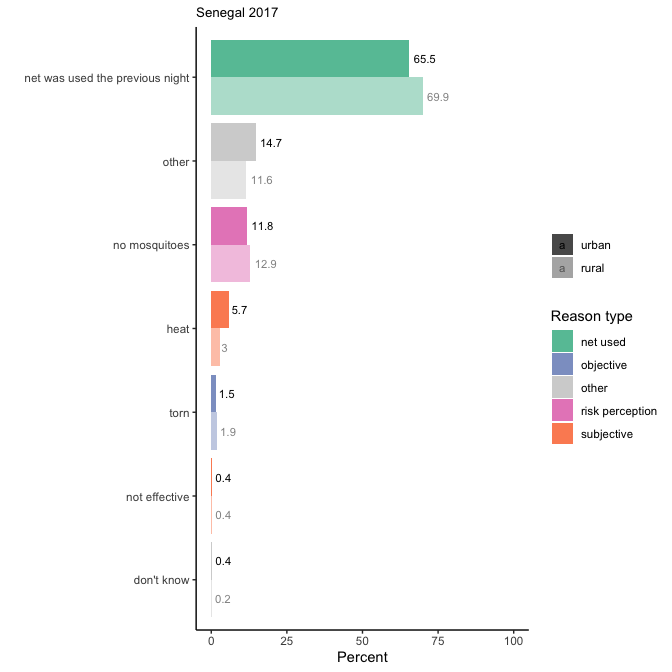


Fig. S20: Reasons nets were not used the previous night, Senegal 2017


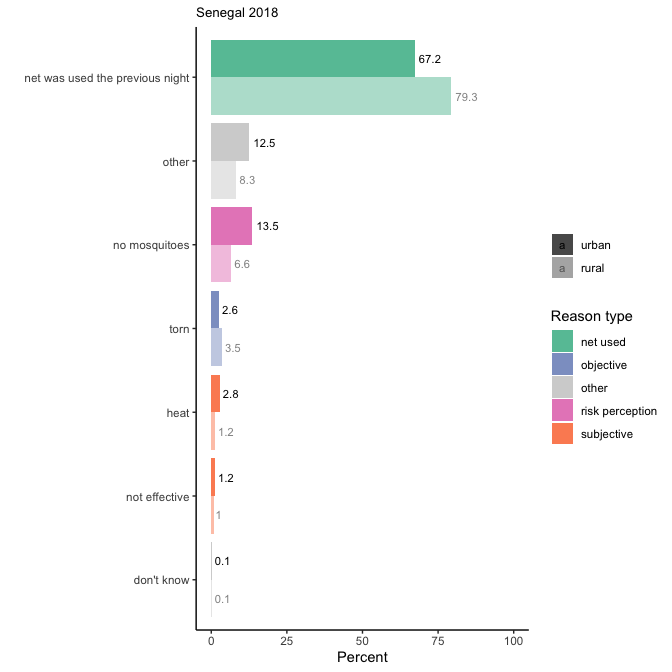


Fig. S21: Reasons nets were not used the previous night, Senegal 2018


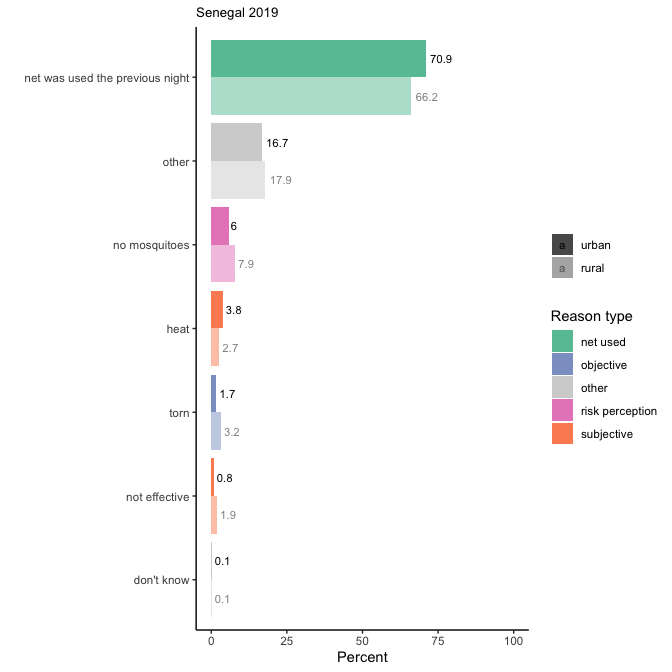


Fig. S22: Reasons nets were not used the previous night, Senegal 2019


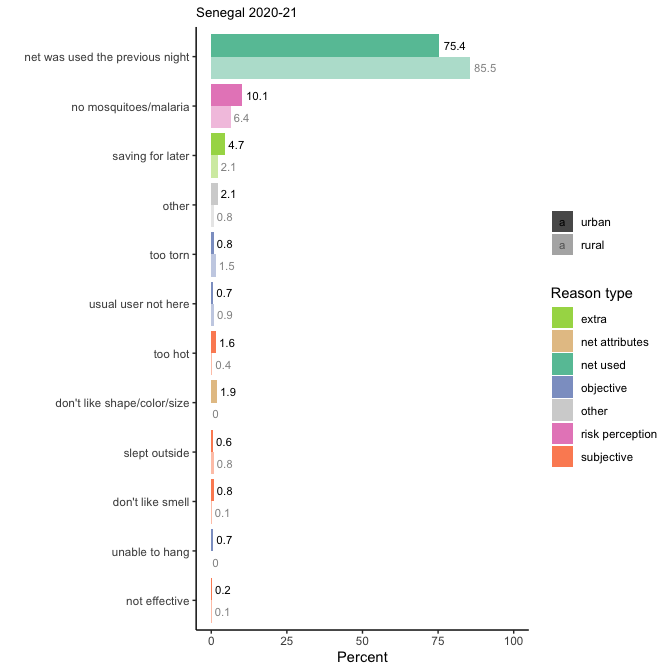


Fig. S23: Reasons nets were not used the previous night, Senegal 2020-2021

### Tanzania


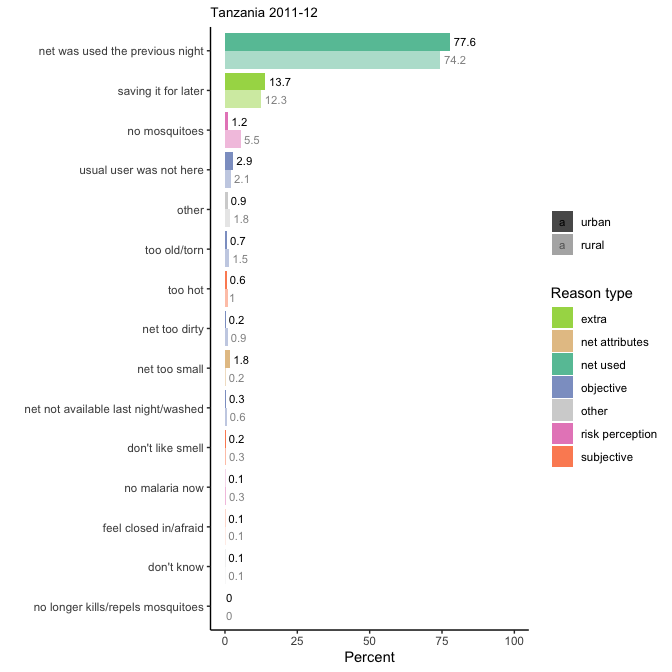


Fig. S24: Reasons nets were not used the previous night, Tanzania 2011-12


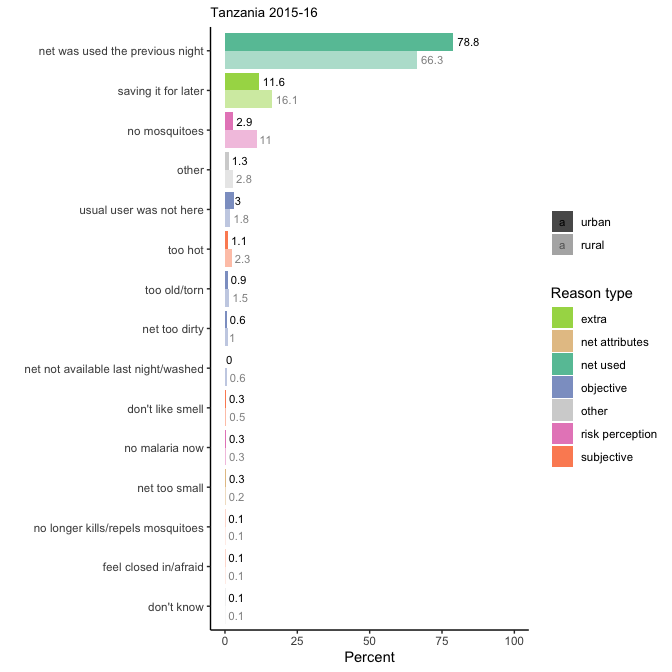


Fig. S25: Reasons nets were not used the previous night, Tanzania 2015


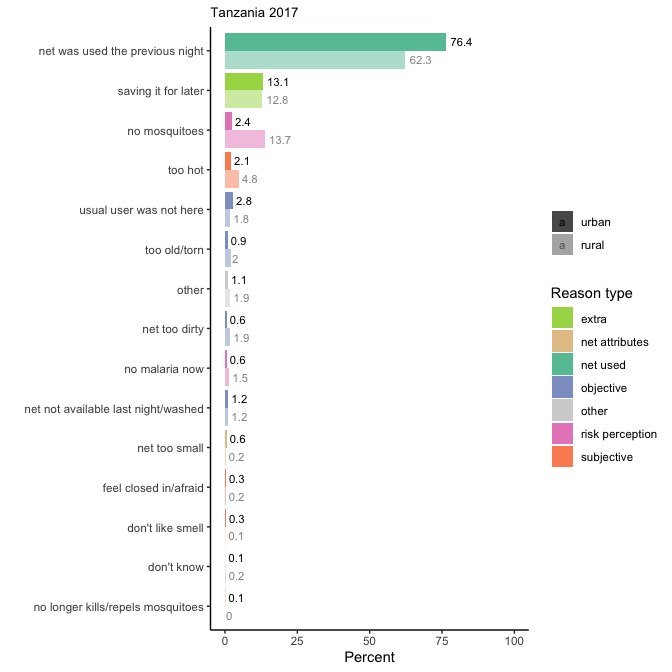


Fig. S26: Reasons nets were not used the previous night, Tanzania 2017-2018

### Uganda


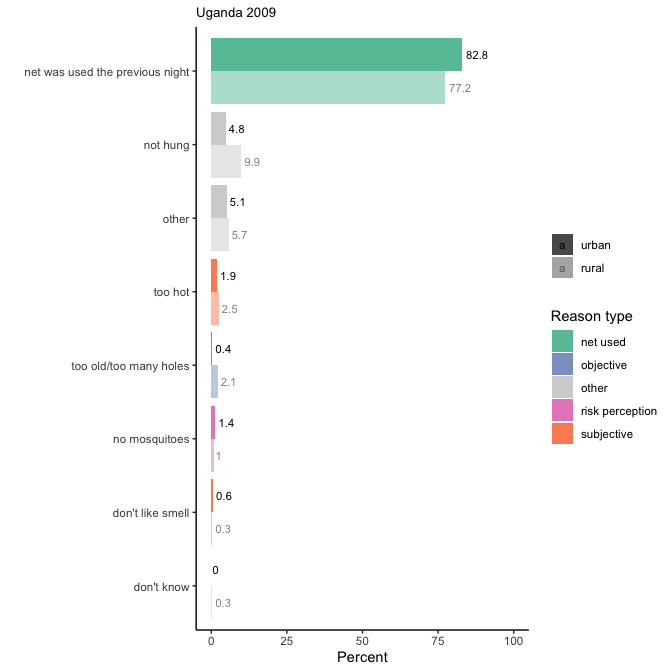


Fig. S27: Reasons nets were not used the previous night, Uganda 2009


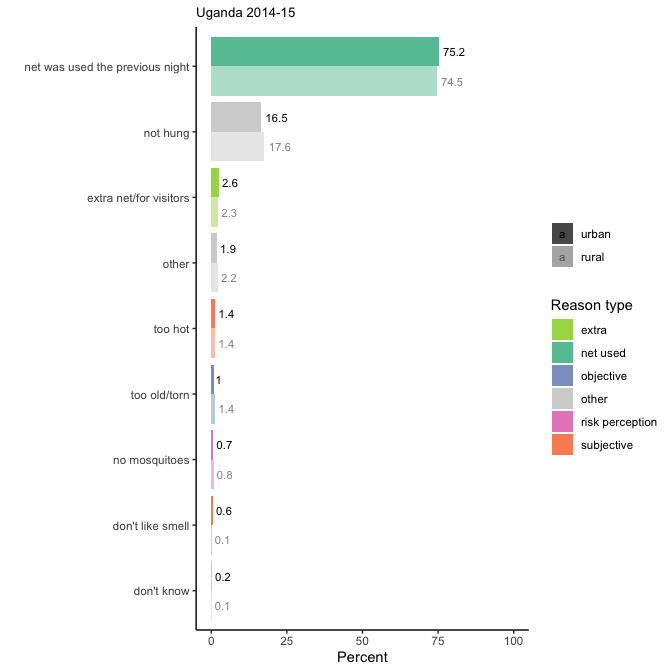


Fig. S28: Reasons nets were not used the previous night, Uganda 2014


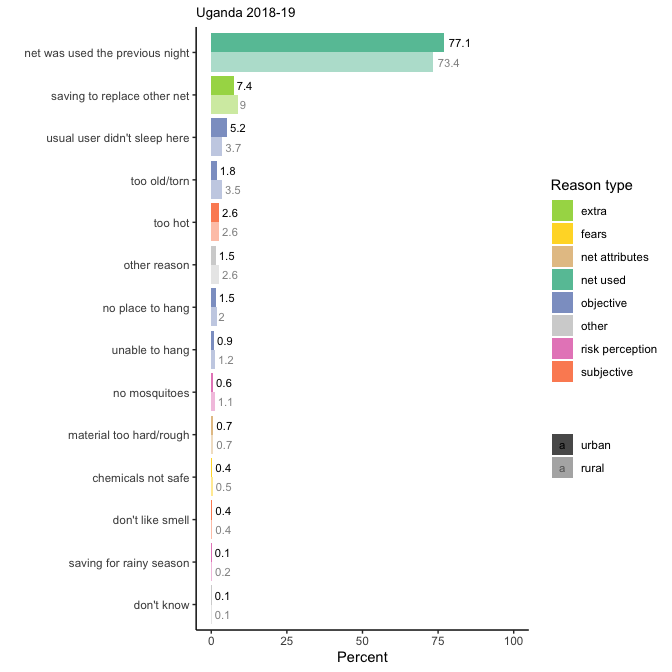


Fig. S29: Reasons nets were not used the previous night, Uganda 2019
